# *Mycobacterium smegmatis* But Not *Mycobacterium avium* subsp. *hominissuis* Causes Increased Expression of the Long Non-Coding RNA MEG3 in THP-1-Derived Human Macrophages and Associated Decrease of TGF-β

**DOI:** 10.3390/microorganisms7030063

**Published:** 2019-02-27

**Authors:** Soroush Sharbati, Faustine Ravon, Ralf Einspanier, Jennifer zur Bruegge

**Affiliations:** 1 Department of Veterinary Medicine, Institute of Veterinary Biochemistry, Freie Universität Berlin, 14163 Berlin, Germany; soroush.sharbati@fu-berlin.de (S.S.); faustine.ravon@ulb.ac.be (F.R.); ralf.einspanier@fu-berlin.de (R.E.); 2 Microbiology, Bioorganic & Macromolecular Chemistry Research Unit, Faculté de Pharmacie, Université Libre de Bruxelles, 1050 Brussels, Belgium

**Keywords:** mycobacteria, long non-coding RNAs, lncRNA, DNA methyltransferases, MEG3, TGF-β, DNMT1, DNMT3b

## Abstract

Pathogenic mycobacteria are able to persist intracellularly in macrophages, whereas non-pathogenic mycobacteria are effectively combated and eliminated after their phagocytosis. It is known that TGF-β plays an important role in this context. Infection with pathogenic mycobacteria such as *Mycobacterium tuberculosis* or *M. avium* leads to production of active TGF-β, which blocks the ability of IFN-γ and TNF-α to inhibit intracellular replication. On the other hand, it is known that the long non-coding RNA (lncRNA) maternally expressed 3 (MEG3) is involved in the regulation of TGF-β. In this study, we show how the infection of THP-1-derived human macrophages with the saprophytic *M. smegmatis* but not with the facultatively pathogenic *M. avium* subsp. *hominissuis* leads to increased MEG3 expression. This is associated with the downregulation of DNA methyltransferases (DNMT) 1 and 3b, which are known to regulate MEG3 expression via promoter hypermethylation. Consequently, we observe a significant downregulation of TGF-β in *M. smegmatis*-infected macrophages but not in *M. avium* subsp. *hominissuis* pointing to lncRNAs as novel mediators of host cell response during mycobacterial infections.

## 1. Introduction

The genus *Mycobacterium* comprises several species, including obligate pathogens such as *M. tuberculosis* (MTB), facultative pathogens such as *M. avium* subsp. *hominissuis* (MAH), and saprophytic species such as *M. smegmatis* (MS), which is usually considered as non-pathogenic. A great variability exists regarding their strategies to persist and multiply in the environment or host organism. Pathogenic members of the genus such as MTB and MAH developed strategies to evade the antimicrobial activities of macrophages and to replicate intracellularly resulting in disease, while MS has only very limited ability to survive in immune cells [1,2,3,4,5].

Identification of the mechanisms used by mycobacteria to subvert immune response is indispensable to understand pathogenesis and to develop strategies for counteracting infection. Over the last few years, several studies reported that mycobacteria influence the expression of regulatory non-coding RNAs (ncRNAs) such as long non-coding RNAs (lncRNAs) affecting host cell response signaling pathways such as autophagy of immune cells [6,7,8,9,10]. Long ncRNAs are distinguished from other non-coding RNAs based on their size of larger than 200 nucleotides. Long ncRNAs function, for example, as protein scaffolds, activators or inhibitors of transcription, antisense RNA, protein decoys, or microRNA (miRNA) sponges [11]. In contrast to miRNAs, studies investigating the role of lncRNAs in mycobacterial infections are just beginning to rise. For example, it was shown that the lncRNA CD244, which is upregulated in MTB infection, acts as an epigenetic inhibitor of TNF-α and IFN-γ expression [12]. The authors were able to show that lncRNA CD244 leads to trimethylation and a more repressive chromatin state at the IFN-γ or TNF-α loci. However, the infection-related function and mode of action of most reported lncRNAs remain to be investigated.

Recently, we identified the participation of the lncRNA maternally expressed 3 (MEG3) in the process of autophagy in macrophages infected with *M. bovis* BCG [6]. In the present study, we focused on the expression of the lncRNA MEG3 in response to other mycobacteria (MS and MAH), as well as the cellular regulation of MEG3 and its function regarding TGF-β expression, a cytokine which is known to play an important role during mycobacterial infection [13,14,15]. Our findings provide novel insight into the regulatory function of lncRNA MEG3 in response to mycobacteria exhibiting differences in virulence, such as the ability to persist intracellularly, and improve our understanding of the mycobacterium–macrophage interplay.

## 2. Materials and Methods

### 2.1. Bacterial Strains and Culture Conditions

*M. smegmatis* mc^2^ 155 (DSMZ No. 43756) and *M. avium* subsp. *hominissuis* strain 104 [16] were cultured on Middlebrook 7H11 (BD Life Sciences, Heidelberg, Germany) agar plates including 10% OADC supplement (BD Life Sciences) and 0.5% glycerol (Carl Roth GmbH, Karlsruhe, Germany) at 37 °C until colonies were visible. Colonies were transferred from plates to Middlebrook 7H9 broth (BD Life Sciences) supplemented with 10% ADC (BD Life Sciences) and 0.05% Tween-80 (Carl Roth GmbH) and grown at 37 °C until the culture reached an optical density (OD_600_) = 1. From this pre-culture, the main culture was inoculated and adjusted to OD_600_ = 0.1 and cultured again at 37 °C until OD_600_ = 1. Bacteria were harvested by centrifugation, quick-frozen in liquid nitrogen, and kept at −80 °C in PBS containing 10% glycerol until used for infection experiments. For quantification of bacteria, the number of colony-forming units was determined by plating serial dilutions on Middlebrook 7H11 agar plates which were incubated at 37 °C until colonies were visible.

### 2.2. Cell Culture

The monocytic cell line THP-1 (DSMZ No. ACC 16) was cultured in RPMI 1640 (Biochrom AG, Berlin, Germany) supplemented with 10% FBS superior (Biochrom AG), 2 mM l-glutamine (Biochrom AG), 1 mM Na-pyruvate (Biochrom AG), gentamycin (10 µg/mL) (Biochrom AG), and 1 mM HEPES buffer (Biochrom AG) at 37 °C in a 5% CO_2_ humidified atmosphere and passaged 2–3 times per week. Cells were used up to passage 20. To perform infection experiments, cells were differentiated into macrophages by stimulation with phorbol-12-myristate-13-acetate (PMA, Sigma-Aldrich, Munich, Germany) 48 h prior to infection. For that purpose, cells were seeded in a 10 µM PMA solution in RPMI including listed supplements but without antibiotics at a density of 1 × 10^6^ cells per well, applying a 1.5-mL volume together with a six-well cell culture plate (Sarstedt AG, Nümbrecht, Germany). After 24 h of PMA stimulation, the stimulus was removed by washing the adherent cell layer with PBS. Medium including listed supplements but without antibiotics was provided for another 24 h before using THP-1-derived macrophages for infection experiments.

### 2.3. Infection Experiments

In total, 1 × 10^6^ THP-1-derived macrophages were infected using 1 × 10^7^ bacteria (multiplicity of infection (MOI) = 10) at 37 °C and 5% CO_2_. Non-infected cells served as a negative control. Samples were taken 30 minutes, 4 h, and 8 h after adding the bacteria to the cells. For the 8 h time point, cells were washed three times with PBS after 4 h of incubation and incubated in fresh media for another 4 h. For RNA extraction, cells were washed three times with PBS, and lysed with RNA lysis buffer (miRVana, Thermo Fisher Scientific, Darmstadt, Germany), and total RNA was isolated according to the manufacturer’s instruction. Each infection experiment was carried out in three biological replicates (*n* = 3). Post-infection times of 30 min and 4 h were selected based on the observations we made in our previous study regarding MEG3 regulation [3]. The additional time point of 8 h was selected to track possible consequences following MEG3 dysregulation.

### 2.4. Expression Analysis Using RT-qPCR

Complementary DNA (cDNA) was synthesized by reverse transcription using the Maxima First Strand cDNA synthesis kit (Thermo Fisher Scientific) as described in the manufacturer’s protocol. Pooled cDNA was taken as a template for testing the primers listed in Appendix A as described earlier [17]. Expression analysis was performed by means of SYBR Green detection chemistry using the SensiMix SYBR Hi-ROX Kit (Bioline GmbH, Luckenwalde, Germany) as described earlier [6] using a PikoReal Cycler (Thermo Fisher Scientific,). Expression was normalized using simultaneously amplified reference genes (*GAPDH*, *SDHA*, *B2M*). The two most stable reference genes were selected after geNorm analysis [18]. The stable expression of the reference genes is shown in Appendix B. The ΔΔCT method was used to calculate the relative fold difference of RNA expression levels compared to the negative control [19]. Data were baseline-corrected by defining the average of respective negative controls as baseline and calculating the ratio (value/baseline) of replicates using GraphPad Prism version 6.00 (GraphPad Software, La Jolla California USA, www.graphpad.com). The presented data reflect the means of three biological and three technical replicates.

### 2.5. Statistical Analysis

Unpaired *t*-tests were conducted to test significant differences between two treatments. Asterisks in figures summarize *p*-values (* *p* < 0.05, ** *p* < 0.01, *** *p* < 0.001) applying GraphPad Prism version 6.00 for Windows (GraphPad Software, La Jolla, CA, USA, www.graphpad.com).

## 3. Results

### 3.1. Infection with M. smegmatis But Not M. avium subsp. hominissuis Leads to Upregulation of the lncRNA MEG3

**Figure 1:**
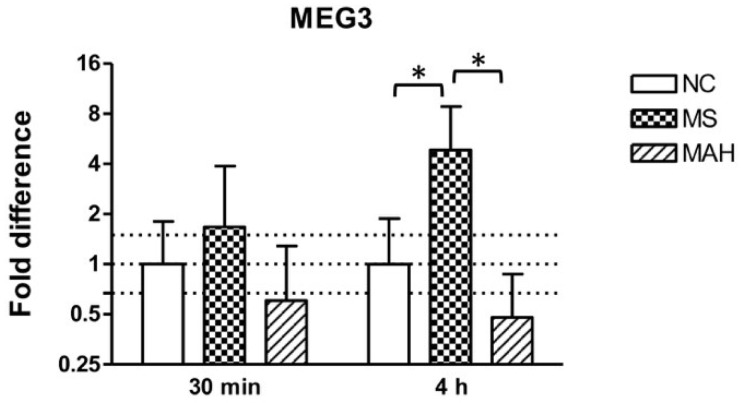
Maternally expressed 3 (MEG3) expression of THP-1-derived macrophages in response to *Mycobacterium smegmatis* (MS, dotted columns) or *M. avium* subsp. *hominissuis* (MAH, dashed columns) compared to the negative control (NC, white columns). Dotted lines indicate the fold differences to negative controls (0.67, 1, and 1.5). The area between the outer dotted lines indicates balanced expression between samples and controls. MEG3 is significantly upregulated by MS 4 h post infection (p.i.) compared to NC and MAH. Asterisks summarize *p*-values (unpaired *t*-test; * *p* < 0.05). Columns show means of three biological replicates (*n* = 3) and bars show the standard deviation.

THP-1-derived macrophages were infected with MS or MAH to analyze MEG3 expression in response to different mycobacteria. As shown in Figure 1, the infection with MS showed clearly increased MEG3 expression 30 minutes post infection (p.i.) (mean fold difference to negative control: 1.68) and even more pronounced and significant upregulation 4 h p.i. (mean fold difference to negative control: 4.84, unpaired *t*-test, *p* < 0.05). In contrast, infection with MAH reduced cellular MEG3 levels 30 minutes (mean fold difference to negative control: 0.6) and 4 h p.i. (mean fold difference to negative control: 0.48).

### 3.2. The Expression of DNA Methlytransferases 1 and 3b Is Downregulated after M. smegmatis Infection

The cellular expression of MEG3 was reported to be regulated by DNA methyltransferase (DNMT) 1 in lung cancer cells [20] and in glioma cells [21], as well as by both DNMT1 and 3b in hepatocellular cancer cells [22]. To follow up if this is also the case in mycobacterial infections of human macrophages, the expression of DNMT1 and DNMT3b was analyzed by RT-qPCR 30 min, 4 h, and 8 h p.i. with MS or MAH.

**Figure 2:**
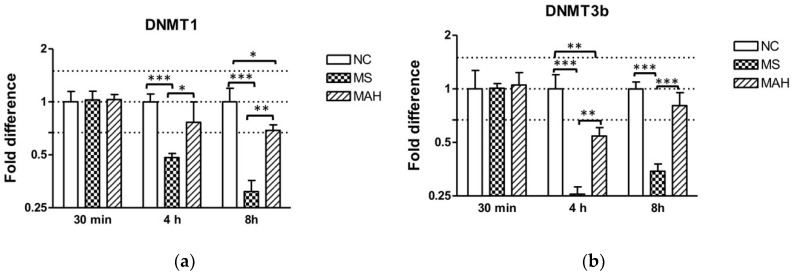
Expression of DNA methyltransferase (DNMT) 1 and 3b in macrophages infected with *M. smegmatis* (MS, dotted columns) and *M. avium* subsp. *hominissuis* (MAH, dashed columns) compared to the negative control (NC, white columns). Dotted lines indicate the fold differences to negative controls (0.67, 1, and 1.5). The area between the outer dotted lines indicates balanced expression between samples and controls. (**a**) Infection with MS caused a clear downregulation of DNMT1 expression compared to the negative control and to MAH 4 h and 8 h p.i. (**b**) DNMT3b expression is significantly reduced by MS 4 h p.i. compared to the negative control and compared to infection with MAH. Asterisks summarize *p*-values (unpaired *t*-test; * *p* < 0.05, ** *p* < 0.01, *** *p* < 0.001). Columns show means of three biological replicates (*n* = 3) and bars show the standard deviation.

As shown in Figure 2a, infection with MS caused highly significant downregulation of DNMT1 expression compared to both the negative control and MAH at 4 h p.i. (mean fold difference to negative control: 0.48, unpaired *t*-test, *p* < 0.001), as well as 8 h p.i. (mean fold difference to negative control: 0.31, unpaired *t*-test, *p* < 0.001), whereas infection with MAH only caused slightly decreased DNMT1 expression 4 h p.i. (mean fold difference to negative control: 0.76) and 8 h p.i. (mean fold difference to negative control: 0.69, unpaired *t*-test, *p* < 0.05). DNMT3b was very pronounced and highly significantly decreased by MS 4 h p.i. (mean fold difference to negative control: 0.26, unpaired *t*-test, *p* < 0.001) and 8 h p.i. (mean fold difference to negative control: 0.34, unpaired *t*-test, *p* < 0.001) compared to the negative control and to infection with MAH. In contrast, MAH only produced slightly decreased expression 4 h p.i. (mean fold difference to negative control: 0.54, unpaired *t*-test, *p* < 0.01) (Figure 2b).

### 3.3. TGF-β Is Downregulated in M. smegmatis-Infected THP-1-Derived Macrophages

**Figure 3:**
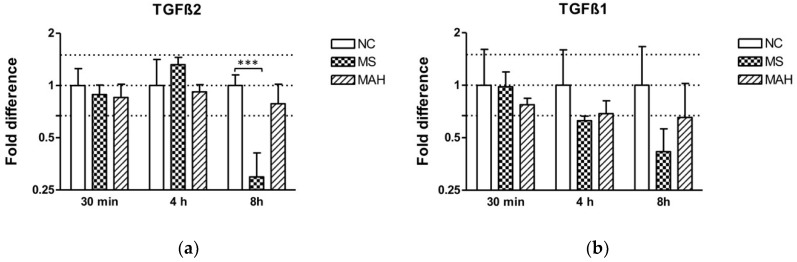
Expression of TGF-β2 and TGF-β1 in macrophages infected with *M. smegmatis* (MS, dotted columns) and *M. avium* subsp. *hominissuis* (MAH, dashed columns) compared to the negative control (NC, white columns), *n* = 3. (**a**) Expression of TGF-β2 is clearly reduced in MS-infected cells 8 h p.i. (**b**) Expression of TGF-β1 is downregulated by MS 8 h p.i. Asterisks summarize *p*-values (unpaired *t*-test; *** *p* < 0.001). Columns show means of three biological replicates (*n* = 3) and bars show the standard deviation.

After showing pronounced downregulation of methyltransferases, we focused our investigations on TGF-β. MEG3 is known to modulate the activity of TGF-β gene expression by forming RNA–DNA triplex structures, thereby binding distal regulatory elements, leading to its transcriptional repression in breast cancer cells [23]. As TGF-β is a crucial regulator of the immune response during mycobacterial infections, we were interested if TGF-β expression is dysregulated in our infection model. Interestingly, we found highly and significantly decreased TGF-β2 expression in MS-infected cells 8 h p.i. (mean fold difference to negative control: 0.29, unpaired *t*-test, *p* < 0.001). In contrast, MAH infections did not cause a significant dysregulation of both TGF-β1 and 2 expression (Figure 3a). Expression of TGF-β1 was downregulated by MS 8 h p.i. (mean fold difference to negative control: 0.42) (Figure 3b).

## 4. Discussion

Several studies reported the impact of mycobacteria on the expression of host cell lncRNAs. However, function and molecular mechanisms of these lncRNAs usually remain unknown [12,24,25]. Furthermore, in many cases, it is unknown if the expression of lncRNAs is dysregulated in a pathogen-specific manner and if dysregulation favors the host or the pathogen. Studies examining the lncRNA expression in response to a choice of pathogens possessing different virulence mechanisms are scarce. However, it was shown that two strains of MTB differing in virulence induced distinct lncRNA expression profiles [8].

As our group recently identified the lncRNA MEG3 as a regulator of autophagy in infections with *M. bovis* BCG belonging to the MTB complex, we were interested if MEG3 expression in macrophages is also affected by facultatively pathogenic or saprophytic mycobacteria possessing differences in the ability to survive in immune cells. MS can be found in normal human genital secretions, as well as in the environment, and is usually not considered a human pathogen. However, there are very few reports on skin or soft-tissue infections, usually occurring under immunosuppressed conditions. It is known that MS is eradicated by macrophages shortly after internalization [2,5]. In contrast, MAH is considered a pathogenic species belonging to the *Mycobacterium avium* complex, frequently causing respiratory illness in immunocompromised patients [26,27]. In addition, cases of *M. avium* infections in immunocompetent patients do occur regularly and are increasing [28,29,30]. MAH is able to invade macrophages and inhibits intracellular killing to ensure its survival and replication [1].

To investigate the expression of lncRNA MEG3 in response to MS or MAH, we infected THP-1-derived macrophages and analyzed MEG3 expression 30 minutes and 4 h p.i. Interestingly, MEG3 expression was significantly induced 4 h p.i. by MS but not MAH. This confirms our observations during *M. bovis* BCG infection [6] and points to a virulence- and species-dependent impact on the expression of MEG3. To investigate the dysregulation of MEG3 expression in more detail, we analyzed the cellular abundance of DNMT1 and 3b messenger RNA (mRNA). These methyltransferases inhibit MEG3 expression via methylation of the MEG3-promotor region, leading to a more repressive chromatin state [20,21,22]. Thus, limited abundance of methyltransferases in MS-infected cells will allow increased expression of the lncRNA MEG3. In accordance with our hypothesis, we found the expression of DNMT1 and 3b markedly and significantly downregulated in MS-infected cells compared to the negative control, but not in MAH-infected macrophages.

In addition, we were interested in the functional aspect of MEG3 upregulation in MS-infected macrophages. MEG3 was recently shown to regulate genes of the TGF-β pathway through formation of RNA–DNA triplex structures [23]. TGF-β was identified as a direct target. Increased expression of MEG3 resulted in significant downregulation of TGF-β. Consistent with the hypothesis that increased MEG3 expression in MS-infected cells leads to decreased TGF-β levels, we found TGF-β to be significantly downregulated in MS but not in MAH-infected cells. TGF-β is recognized as an anti-inflammatory cytokine and has a variety of inhibitory effects including downregulation of macrophage activity and function [31,32]. Several studies showed that TGF-β is produced by macrophages in response to pathogenic mycobacteria such as MTB and *M. avium*, promoting the intracellular persistence and growth of these pathogens [15,33,34]. Infection with MTB or *M. avium* leads to production of active TGF-β, which blocks the ability of either IFN-γ or TNF-α to inhibit intracellular replication [33]. It was shown that TGF-β inhibits the capability of IFN-γ to induce the release of reactive nitrogen intermediates [31]. Neutralization of TGF-β results in increased bacterial killing [33]. These studies elucidate TGF-β as an important mediator of macrophage reactivity to intracellularly persisting mycobacterial pathogens. 

In our study, we observed virulence-dependent regulation of MEG3 expression which corresponded to regulation of DNMT1 and 3b, pointing to the control of MEG3 expression by imprinting [20,21,22]. As already examined in a different context [23], it can be assumed that MEG3 regulates TGF-β also during macrophage infection with the non-pathogenic MS, which remains to be examined experimentally. This seems to represent the normal protective host cell response for eradicating phagocytosed bacteria. Depending on their genetic repertoire, pathogenic mycobacteria seem to have evolved active mechanisms that interfere with the MEG3-mediated downregulation of TGF-β signaling, facilitating their intracellular persistence in host macrophages. Our findings deepen the understanding of mycobacterial pathogenesis and provide novel insights into the regulatory function of lncRNAs during mycobacterial infection of human macrophages.

## Figures and Tables

**Figure A1:**
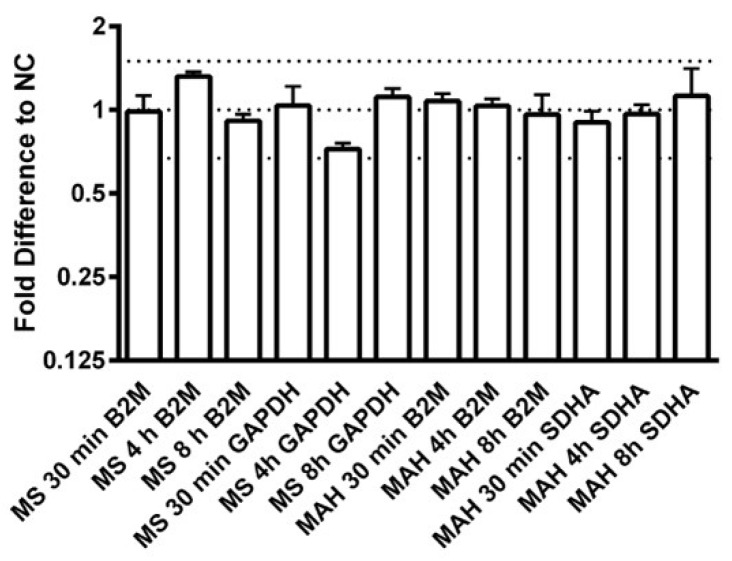
Stable expression of reference genes (*B2M*, *GAPDH*, and *SDHA*) after infection with *M. smegmatis* (MS) or *M. avium* subsp. *hominissuis* (MAH). The fold difference to the negative control (NC) is shown. Columns show means of three biological replicates (*n* = 3) and bars show the standard deviation.

## Appendix A

**Table A1:** Oligonucleotides used for RT-qPCR.

Gene Name	Forward 5′–3′	Reverse 5′–3′	Annealing Temperature
*MEG3*	CAGCCAAGGTTCTTGAAAGG	TTCCACGGAGTAGAGGCAGT	60 °C
*DNMT1*	GAATCAGTTATGTGACTTGGAAACC	CTAGACGTCCATTCACTTCCC	60 °C
*DNMT3b*	CCCATTCGAGTCCTGTCATTG	TTGATATTCCCCTCGTGCTTC	62 °C
*TGF-β1*	CAGCAACAATTCCTGGCGATA	AAGGCGAAAGCCCTCAATTT	60 °C
*TGF-β2*	CCCCGGAGGTGATTTCCATC	CAACTGGGCAGACAGTTTCG	60 °C
*B2M*	GTGCTCGCGCTACTCTCTCT	GGATGGATGAAACCCAGACA	60 °C
*GAPDH*	CCATCTTCCAGGAGCGAGAT	CTAAGCAGTTGGTGGTGCAG	60 °C
*SDHA*	TGGGAACAAGAGGGCATCTG	CCACCACTGCATCAAATTCATG	60 °C
